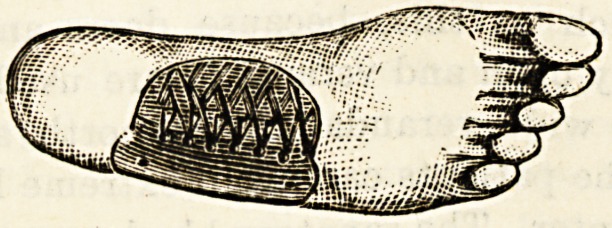# New Appliances and Things Medical

**Published:** 1903-02-14

**Authors:** 


### NEW APPLIANCES AND THINGS MEDICAL.

**[We shall be glad to receive at our Office, 28 & 29 Southampton Street, Strand, London, W.C., from the manufacturers, specimens of all new preparations and appliances which may be brought out from time to time.]**

### DR. DAVIES' TRIPLE SPRING ARCH SUPPORT.

(The Spring Arch Support Company, Limited, 380 Hackney Road, London, N.E.)

Agents: The London Shoe Co., 123 & 125 Queen Victoria Street, E.C.

The instep support, or valgus pad, illustration of which is appended, is an ingenious modification of Dr. Davies' original steel comb support. It is made of three separate toothed springs, with the teeth cut in three directions and interlaced so as to form an elastic and single spring of great strength and resiliency. This triple spring is covered with kid, and held in position by an elastic strap. It is a very practical form of instep support for flat foot.

### KARNOID.

(Karnoid, Limited, 6 and 7 Stonecutter Street, Ludgate Circus, London, E.C.)

Karnoid is a dry powder containing rather more than 50 per cent. of nitrogenous compounds, a small percentage of fat, and a considerable percentage of extractives. Over 50 per cent. of the powder is soluble in cold water, and when prepared according to the directions affords a highly nutritious and agreeable cup of beef tea, containing a far higher proportion of nutritive material than is the case with beef tea prepared by the ordinary methods. Karnoid is obtained from the flesh of beef, veal, chicken, turtle, etc., by a special process without the addition of chemical preservatives, and it constitutes a valuable addition to the list of albuminoid invalid specialities. It can be tolerated and digested by the most delicate and sensitive stomach, and readily assimilated in cases of debility.

## Figures and Tables

**Figure f1:**